# Deterministic and stochastic interventions in reducing drug–drug interactions in inappropriate prescribing: A systematic review

**DOI:** 10.1371/journal.pone.0356883

**Published:** 2026-09-17

**Authors:** Muhammad Fahmi Ahmad Zuber, Nur Aishah Che Roos, Ruzanna Mat Jusoh, Nurulhuda A Manaf

**Affiliations:** 1 Institute for Cybersecurity and Electronic Systems, Centre for Defence Foundation Studies, National Defence University of Malaysia (UPNM), Kuala Lumpur, Malaysia; 2 Faculty of Medicine and Defence Health, National Defence University of Malaysia (UPNM), Kuala Lumpur, Malaysia; 3 Department of Defence Science, Faculty of Defence Science and Technology, National Defence University of Malaysia (UPNM), Kuala Lumpur, Malaysia; 4 Department of Mathematics, Centre for Defence Foundation Studies, National Defence University of Malaysia (UPNM), Kuala Lumpur, Malaysia; Federal University of Rio Grande do Sul Faculty of Medicine: Universidade Federal do Rio Grande do Sul Faculdade de Medicina, BRAZIL

## Abstract

**Background:**

Drug–drug interactions (DDIs) remain a major contributor to preventable patient harm, particularly in the context of polypharmacy. Over the past two decades, interventions to mitigate inappropriate prescribing have evolved from deterministic, rule-based clinical decision support toward increasingly complex data-driven and stochastic models. However, the extent to which these methodological advances translate into improved clinical safety remains unclear.

**Methods:**

We conducted a systematic review in accordance with PRISMA 2020 guidelines, guided by the SPIDER framework. PubMed, Scopus, ScienceDirect, and IEEE Xplore were searched from inception to October 2025 for primary studies evaluating computational or clinical decision support interventions aimed at reducing DDIs or inappropriate prescribing. Eligible studies included deterministic rule-based systems, ontological frameworks, and artificial intelligence-driven predictive models. Risk of bias was assessed using the Prediction Model Risk of Bias Assessment Tool, extended with artificial intelligence-specific considerations (PROBAST+AI). Due to heterogeneity in study designs and outcome measures, findings were synthesized narratively.

**Results:**

Ten studies met the inclusion criteria. Earlier interventions predominantly employed deterministic approaches focused on workflow optimization, alert management, and policy enforcement, demonstrating modest improvements in prescribing processes but inconsistent links to patient-level outcomes. More recent studies applied stochastic and generative models using high-dimensional clinical datasets to predict DDIs, reporting strong internal performance metrics. However, PROBAST+AI assessment identified a consistently high risk of bias in the analysis domain for AI-driven studies, primarily due to limited external validation, insufficient calibration reporting, and unclear handling of overfitting and data leakage.

**Conclusions:**

While stochastic and generative models offer enhanced predictive capacity for DDI detection, current evidence does not demonstrate a proportional improvement in clinically reliable decision support. Deterministic systems provide transparency and safety constraints but lack adaptability to patient-specific contexts. Future interventions must prioritize hybrid architectures that integrate explainable rule-based guardrails with rigorously validated stochastic models to ensure that methodological complexity yields reproducible gains in patient safety.

## Introduction

Medical error remains a major global patient-safety concern, encompassing diagnostic errors, treatment errors, preventive errors, and system or communication failures. The landmark report To Err Is Human [1] highlighted the scale of preventable harm in healthcare, and subsequent initiatives by organizations such as the World Health Organization have reinforced medication safety as a priority domain. Among medication-related errors, inappropriate prescribing has received particular attention because of its direct link to adverse drug events and hospitalizations. One increasingly prominent driver of inappropriate prescribing is drug-drug interaction (DDI) risk arising from polypharmacy, especially in aging populations with multimorbidity. As patients accumulate medications across specialties and care settings, clinicians face escalating cognitive and informational demands, positioning DDI management as a critical and complex subset of medical error research.

Polypharmacy creates a combinatorial explosion in the state space of patient outcomes. As the number of prescribed medications (*n*) increases, the potential for pairwise interactions grows at a rate of $\frac{n(n-1)}{2}$, the standard combinatorial formula for selecting all possible pairs from n items. This figure represents the number of searches to fully map out interaction of two drugs, for total of n drugs. Historically, the burden of navigating this high-dimensional risk landscape fell upon human cognition, supported by static, rule-based knowledge bases. However, the rapid increase in the publication of medical literature and the complexity of patient comorbidities lead to information overload for human-only evidence synthesis.

The literature on inappropriate prescribing is presently characterized by a clear separation of research domains. Within clinical operations research, DDI mitigation is typically framed as a workflow optimization problem, where improvements are pursued through alert systems, prescribing policies, and mechanisms designed to calibrate the “human-in-the-loop” within clinical decision processes [2,3]. In contrast, the computational biology and medical artificial intelligence literature approaches the same problem as a predictive modeling task. Here, interaction risk is inferred from large-scale biomedical data using increasingly sophisticated architectures, including graph neural networks (GNNs) [4] and, more recently, generative pre-trained transformer (GPT)-based systems [5]. Previous overviews suggest that although Clinical Decision Support Systems (CDSS) can improve process-level prescribing compliance [6], the causal relationship between these improvements and measurable patient safety outcomes remains inconsistent.

Existing systematic reviews have examined a range of interventions aimed at reducing inappropriate prescribing [7] and DDI-related harm [8], most commonly focusing on CDSS deployment [9], educational initiatives [10], pharmacist-led medication review [11], and policy-level prescribing controls [12]. These syntheses generally report improvements in intermediate process indicators, such as guideline adherence [13] or alert acknowledgment [14], while consistent reductions in patient-level adverse outcomes remain uncertain [15]. At the same time, the methodological landscape is evolving rapidly. Newer approaches, including graph-based learning [16], multimodal clinical data integration [17], and large language model-driven agents [18] are emerging at a pace that traditional evidence syntheses struggle to capture. Moreover, existing reviews tend to examine either health-system interventions [19] or computational prediction models [20] in isolation, leaving an analytical gap between workflow-based prescribing optimization and algorithmic decision intelligence.

Therefore, this systematic review seeks to address this gap by synthesizing evidence across policy-level interventions, clinical operational systems, and emerging AI-driven approaches to DDI mitigation. The review is guided by the following research questions:

- What models or tools have been developed to reduce DDIs in managing inappropriate prescribing?
- How do these models or tools measure the reduction of DDI risk?
- Does the more complex artificial intelligence (AI)-driven model have better performance versus simpler policy-driven models?

## Methodology

### Protocol and registration

This review was conducted in accordance with the Preferred Reporting Items for Systematic Reviews and Meta-Analyses (PRISMA) 2020 statement [21] and the checklist is included as supporting information, S1 Table. The present review protocol is registered on the International Platform of Registered Systematic Review and Meta-analysis Protocols (INPLASY) with registration ID INPLASY202630031 [22]. No significant amendments were made to the protocol during the review process.

### Eligibility criteria

Eligibility criteria were guided by the Sample, Phenomenon of Interest, Design, Evaluation, Research type (SPIDER) framework [23].

1. **Sample:** No restrictions were applied to the type of dataset. Real-world clinical, simulated population, and computational datasets were eligible for inclusion.
2. **Phenomenon of Interest:** Studies addressing prescription errors were included, with a specific focus on DDIs. Studies examining other types of medical error (e.g., dispensing or administration errors) were excluded.
3. **Design:** Studies that include the use or development of computational, statistical, or algorithmic models or tools to optimize safe prescribing. Eligible approaches included, but were not limited to, rule-based systems, artificial intelligence (AI)-driven models, Bayesian models, knowledge graphs, and hybrid or ensemble systems. Studies in which models or tools were used exclusively for diagnostic purposes were excluded.
4. **Evaluation:** Included studies were required to report an evaluation component, such as improvements in prescribing accuracy or appropriateness, reductions in prescribing errors, performance metrics, or model validation.
5. **Research Type:** We included primary studies describing the development, validation, or application of computational models, as well as conference proceedings reporting validated computational models. We excluded publications without empirical data, commentaries, grey literature, systematic reviews or meta-analyses, studies lacking model validation or application, duplicate studies, and studies for which the full text was unobtainable.

### Information sources and search strategy

A structured search strategy was developed using predefined keywords and Boolean operators to identify relevant studies. The search was conducted from database inception to 9 March 2026 across four electronic databases: PubMed, Scopus, ScienceDirect, and IEEE Xplore. A few databases, including Embase, Cochrane Central Register of Controlled Trials (CENTRAL), and Web of Science, along with regional databases such as the Index Medicus for the South-East Asia Region (IMSEAR) and China National Knowledge Infrastructure (CNKI), were not searched due to access restriction.

The search string combined terms across four conceptual domains: (1) inappropriate or erroneous prescribing, (2) drug-related interactions and polypharmacy, (3) optimization or improvement outcomes, and (4) computational or clinical decision support approaches. The final search strategy was formulated as follows:

(“Inappropriate prescri*” OR “Medication error” OR “prescri* error reduction” OR “Prescribing error”)
AND (“Drug-Drug Interaction” OR Polypharmacy OR “Drug Interaction”)
AND (Optimization OR Optimi* OR Improve* OR Reduc*)
AND (“Clinical decision support” OR CDSS OR “Electronic prescri*” OR e-prescri* OR “Decision support system” OR “Predictive model” OR “Rule based” OR “Guideline based” OR Algorithm OR “Artificial intelligence” OR AI OR “Machine learning” OR “Deep learning” OR “Data-driven model” OR “Automated system” OR “Hybrid model” OR “Medica* Pathway”)

The search strategy was adapted as necessary to meet the syntax requirements of each database.

### Selection and data collection process

All retrieved records were imported into Rayyan artificial intelligence (Rayyan-AI) [24] for reference management, deduplication, and screening. Duplicate records were automatically identified and removed, followed by title and abstract screening conducted independently by two reviewers (MF and NA) within the Rayyan platform. Rayyan’s AI-assisted features were not used in decision-making; final inclusion and exclusion decisions were made by the reviewers.

Full-text articles were retrieved for all potentially eligible studies and independently assessed for inclusion. Any disagreements were resolved through discussion, with arbitration by a third reviewer (NH) when necessary. Data extraction was performed using a standardized data extraction form, capturing study characteristics including study design, sample size, model architecture, and reported outcome measures.

### Data items and effect measures

We extracted variables related to the intervention type (Deterministic vs. Stochastic), dataset origin (Clinical vs. Synthetic), and performance metrics. Due to the heterogeneity of the studies, we analyzed any reported effect measures which included alert acceptance rates and error reduction percentages for clinical studies, and Area Under the Curve (AUC), Jaccard similarity, and Root Mean Square Error (RMSE) for computational models.

### Study risk of bias assessment

Risk of bias was assessed using the Prediction Model Risk of Bias Assessment Tool (PROBAST) [25], extended with artificial intelligence–specific considerations (PROBAST + AI). The assessment framework was structured according to the Population, Index model(s), Comparator model(s), Outcome(s), Timing, and Setting, and intended use of the prediction model (PICOT) elements.

Consistent with the PROBAST guidance, we evaluated risk of bias across the domains of Participants (Population), Predictors (Index and Comparator models), Outcomes, and Analysis. Particular emphasis was placed on AI and ML-specific sources of bias, including overfitting, inappropriate handling of missing data, data leakage between training and validation sets, and adequacy of model validation.

The PROBAST+AI framework comprises four domains: Participants and Data Source, Predictors, Outcome, and Analysis. In the present review, the three most critical domains considered for overall risk of bias judgement were Participants and Data Source, Predictors, and Analysis, as these domains are most directly associated with bias arising from dataset representativeness, feature construction, and model development and validation processes in artificial intelligence studies. Adhering to the PROBAST guideline, overall risk of bias was assigned according to the worst-domain-anywhere principle: a study was rated as high risk overall if any single domain was rated high, as low risk overall if and only if all rated low.

### Certainty assessment

A formal certainty-of-evidence assessment framework was not applied. This decision was made because the included studies reported heterogeneous outcomes spanning clinical process indicators and computational performance metrics, which do not represent a single comparable clinical endpoint. Instead, the overall strength and reliability of the evidence were interpreted qualitatively in conjunction with the PROBAST+AI risk-of-bias assessment and the methodological characteristics of the included studies.

### Synthesis methods

We performed a narrative synthesis grouped by the topological nature of the intervention: Clinical-Policy, Ontological, and Stochastic/Generative. Statistical meta-analysis was not feasible due to the disjoint nature of the outcome metrics (e.g., alert fatigue vs. algorithmic accuracy).

Classification of intervention type was based on the underlying decision-making mechanism reported by each study’s authors, rather than on clinical domain or stated aim. Studies were categorized as Deterministic when the system output was generated by fixed, rule-based logic with no learned or probabilistic estimated parameters. Studies were categorized as Ontology-based when DDI detection operated as deterministic reasoning over a formally structured knowledge base (e.g., UMLS- or RxNorm-derived class hierarchies), representing a structured subtype of deterministic architecture rather than an independent mechanism. Studies were categorized as Stochastic/Generative when model output was derived from learned parameters trained on data, yielding probabilistic or pattern-completion predictions.

Deterministic systems generate outputs using fixed, pre-programmed logic with no learned or probabilistic estimated parameters. This category includes simple rule-based alert systems (e.g., ‘If Drug A and Drug B are prescribed, trigger an alert’). as well as more complex Ontological Frameworks. While ontologies map clinical data into highly structured, semantic hierarchies, interaction detection still operates as a deterministic, binary lookup without learned probabilistic estimation.

In contrast, Stochastic and Generative systems generate probabilistic outputs derived from learned parameters trained on clinical or synthetic datasets. Rather than relying on hard-coded rules, these models (including deep learning networks, collaborative filtering, and Large Language Models) infer interaction risks dynamically, treating drug interaction detection as a pattern-recognition or predictive task within a high-dimensional space. Notably, some recent interventions employ Hybrid Architectures, leveraging deterministic ontologies to set safety boundaries while utilizing stochastic models to navigate complex patient variables within those constraints. For classification purposes in this review, studies were categorized based on their primary mechanism of interaction detection.

Due to the heterogeneity in intervention design, dataset origin, and reported outcome measures, a quantitative meta-analysis was not performed. The included studies reported fundamentally different evaluation metrics, ranging from clinical indicators such as reductions in prescribing errors or alert fatigue to computational performance metrics including AUC, Jaccard similarity, and RMSE. As these measures represent different constructs and cannot be meaningfully aggregated into a single effect estimate, results were synthesized using a structured narrative approach. To facilitate interpretation and explore potential sources of heterogeneity, studies were grouped narratively according to the type of intervention, namely Clinical-Policy systems, Ontological frameworks, and Stochastic or Generative computational models. Comparisons across these categories were used to identify methodological differences and patterns in reported outcomes.

Sensitivity analysis was not conducted because the limited number of included studies and the heterogeneity of outcome measures precluded meaningful robustness testing.

## Results

### Study selection

The database search identified a total of 283 records from four electronic databases: PubMed (n = 42), Scopus (n = 18), ScienceDirect (n = 192), and IEEE Xplore (n = 31). After removal of duplicate records (n = 9), 274 records were screened by title and abstract.

During initial screening, 153 records were excluded for the following reasons: inappropriate research type, including systematic reviews or meta-analyses (n = 55), publications without empirical data (n = 44), grey literature (n = 8), lack of validation or application of a computational model (n = 16); ineligible phenomena such as dispensing or administration processes (n = 20) or other types of medical error (n = 8); diagnostic or monitoring-focused designs (n = 15); and policy or management–focused studies (n = 1). For details, see supporting information S1 File.

A total of 121 records were sought for full-text retrieval, of which two could not be retrieved due to the unavailability of the full article. The remaining 119 full-text articles were assessed for eligibility, resulting in the exclusion of 109 studies for reasons stated above and listed in supporting information S2 File.

**Fig 1:**
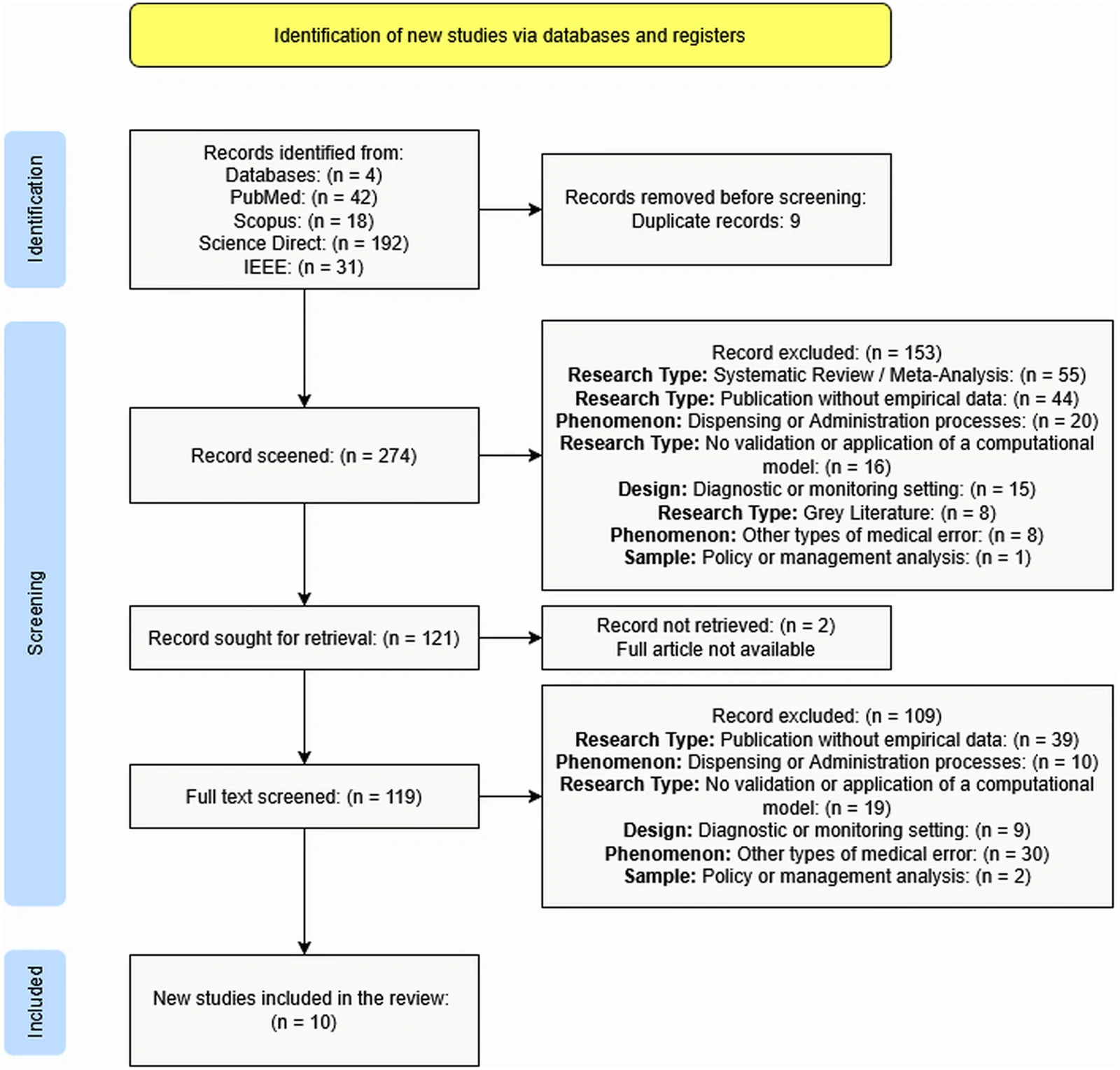
PRISMA flow diagram illustrating the study identification, screening, eligibility assessment, and inclusion process.

Ultimately, 10 studies met all eligibility criteria and were included in the final review. The full screening process are summarized in Fig 1.

### Included studies

Ciampi et al. (2022) [26] applied the Tutor, Checker, Advisor model, using ambient intelligence, reinforcement learning, deep learning, and NLP in home care for elderly patients to reduce omission, dosage/timing errors, and drug–drug interactions, achieving a high F1-score, precision, and accuracy compared with traditional home care. Dou et al. (2024) [27] implemented Shennong-Agent, a large language model, in clinics for 1,000 real-world cases to automate pharmaceutical care and mitigate dose and DDI errors, with performance evaluated by multi-turn expert scoring and comparisons to AI tools including HuataoGPT-II and GPT-4, though limited by potential hallucinations. Saiyed et al. (2019) [28] used the MetroHealth / CaroMont Drug Alert system in hospitals to reduce alert fatigue in EHRs, targeting DDI, DAI, duplicate doses, and TPN alerts, and validated improvements in workflow efficiency against out-of-the-box EHR alerts. Chu (2004) [29] implemented a CDSS in a pilot hospital processing 105,000 prescriptions/day to reduce prescription errors and ADEs through wired and wireless point-of-care e-prescription systems, with qualitative usability evaluation compared to paper-based CPOE. Nuckols et al. (2015) [30] employed CPOE simulation in acute care inpatients to estimate societal savings and QALYs, focusing on allergy- and DDI-related errors with literature-based validation. Mahmud et al. (2024) [31] developed MEDERED, an AI system for smart diagnosis and medication prescription in general patients, addressing allergy, ADE, and DDI errors, with learning outcomes guiding quality-of-care improvements and prototype design for patient safety. Calvo-Cidoncha et al. (2022) [32] designed OntoPharma, an ontology-based CDSS, for hospital medication alerts to reduce dose, DDI, renal, and allergy errors, analyzing 34,938 ontology classes integrated with CPOE via REST API and validated over 1.5 years. Ghibelli et al. (2013) [33] applied INTERcheck, a guideline-based system in an acute geriatric ward for elderly patients with multimorbidity, to reduce PIMs, DDIs, and anticholinergic burden, showing a significant reduction in error rates compared to baseline care. Zomorodi et al. (2024) [34] developed RECOMED, a deep learning and NLP system for global medication and disease profiles, recommending drugs and dosages with high accuracy and evaluated using accuracy, sensitivity, and hit rate metrics. Li et al. (2024) [35] implemented StratMed, a network model on the MIMIC-III ICU dataset, to improve accuracy and safety of drug recommendations, addressing DDI and recommendation sparsity, assessed via F1-score, Jaccard, and HR@k, with cross-validation and computational complexity considerations.

The full data extraction is summarized in Table 1.

**Table 1:** Characteristic of included studies.

Author	Population	Model Name	Model Class	Aims	Medical Error	Scores	Outcomes	Comparator	Validation	Limitation	Data Source
Chu, S. (2004, June)	105,000 prescriptions/day; small pilot hospital	Null	CDSS (D)	Implementing wired and wireless point-of-care ePrescription systems with decision support	Prescription errors, ADEs (wrong drug, dose, interactions)	Qualitative usability ratings; reported reductions in prescription errors	Paper-based / wired CPOE	Technology transition	Qualitative evaluation	New Zealand Ministry of Health data; hospital pilot implementation	Null
Ghibelli, S. et al. (2013)	Two acute geriatric patient multimorbidities	INTERcheck	Guideline-based (D)	Reduce PIMs, DDIs, and anticholinergic burden	PIM, DDI, Anticholinergic burden	Null	Significant reduction in PIMs and severe DDIs	Baseline care (implied)	Clinical profile review	Null	Electronic prescription database
Nuckols, T. K. et al. (2015)	Acute Care Patients	CPOE Simulation	CPOE (D)	Estimate societal savings from CPOE	Allergies, DDI	QALYs lost per fatal ADE	Reduction in pADEs	Without CPOE	Literature-based simulation	Model assumptions	Literature, surveys
Saiyed, S. M. et al. (2019)	MHS: 1.2M outpatient visits; CH: 45 + care sites, 500 staff physicians	MetroHealth / CaroMont	Drug Alert (D)	Reduce alert fatigue and optimize medication-related alerts in EHR systems	DDI, DAI, Duplicate, Dose, TPN	MHS: 38 /100, CH: 51 /100	Reduces irrelevant alerts, improves workflow efficiency, supports safer prescribing	Out-of-box EHR alerts before optimization	System-level optimization and reporting	Standardized drug alert reports, EHR logs	Null
Ciampi, M. et al. (2022)	Elderly Home care	Tutor, Checker, Advisor	Ambient Intelligence, RL, DL, Natural Language Processing (S)	Reduce omission, dosage/timing errors, and drug-drug interactions (DDI)	Dose, Timing, DDI	f1: 0.93, Precision: 0.91, Accuracy: 0.94, Recall: 0.94	Null	Traditional Home	Performance score	Reliance on sensor accuracy; OCR packaging errors	DrugBank, medical reports, and real-time data from home sensors
Calvo-Cidoncha, E., et al. (2022).	Hospital Alerts 2020–2021	OntoPharma	Ontology-based (D)	Design/develop ontology CDSS for error reduction	Dose, DDI, Renal, Allergy	34,938 classes	Integrated with CPOE via REST API	Relational databases	Analysis of alerts over 1.5 years	Null	Protégé-OWL ontologies
Dou, Y., et al. (2024)	1,000 real-world clinical cases	Shennong-Agent	LLM (S)	Automate pharmaceutical care	Dose, DDI	Accuracy: 4.5, Safety: 4.6, Comprehensive: 4.9, Practicality: 4.3, Patient Compliance: 4.5, Redundancy: 4.1	Null	HuataoGPT-II, Clinfo.Ai, medical.chat, GPT4, Qwen	Multi-turn expert review and scoring	Potential for LLM hallucinations/ the need for continuous updates	Prescription data
Mahmud, M. et al. (2024)	Health facilities	MEDERED	AI (S)	Smart diagnosis and medication prescription	Allergy, ADE, DDI		Intelligent re-engineering for quality care		Prototype architectural design	Integration factors in patient safety	PubMed & MHR data
Zomorodi, M. et al. (2024)	Global research; 2964 patient profiles	RECOMED	DL, NLP (S)	Recommend drugs and dosages with high accuracy	DDI, Dosage	CUR (User Rating)	Generates prescriptions similar to real-life cases	Matrix factorization	Knowledge-based system rules	AI trust & ethical issues	Druglib.com & drug databases
Li, X. et al. (2024)	MIMIC-III (46,520 patients)	StratMed	Network Model (S)	Improve accuracy and safety of drug recommendation	DDI / Recommendation sparsity	f1: 0.7011, Precision: 0.742 Jaccard: 0.5401	Reduction in DDI rate in recommendations	GAMENet, SafeDrug, RETAIN	Cross-validation on MIMIC-III	Computational complexity	MIMIC-III Database

**Acronyms used in the table:** Model Class categories are denoted as *D – Deterministic Model and *S – Stochastic Model. ADE/ADEs - Adverse Drug Event(s); AI – Artificial Intelligence; CDSS – Clinical Decision Support System; CH – CaroMont Health; CPOE – Computerized Physician/Provider Order Entry; CUR – User Rating metric as defined by Zomorodi et al. (2024); DAI – Drug Allergens; DDI/DDIs - Drug–Drug Interaction(s); DL – Deep Learning; EHR – Electronic Health Record; f1 - F1 Score, the harmonic mean of precision and recall; GPT – Generative Pre-trained Transformer; LLM – Large Language Model; MHR – Medical Health Record(s); MHS – MetroHealth System; MIMIC-III – Medical Information Mart for Intensive Care III; NLP – Natural Language Processing; OCR – Optical Character Recognition; OWL – Web Ontology Language; pADE/pADEs – Preventable ADE(s); PIM/PIMs - Potentially Inappropriate Medication(s); QALY/QALYs - Quality-Adjusted Life Year(s); REST API – Representational State Transfer Application Programming Interface; RL – Reinforcement Learning; TPN – Total Parenteral Nutrition.

### Risk of bias in studies

**Fig 2:**
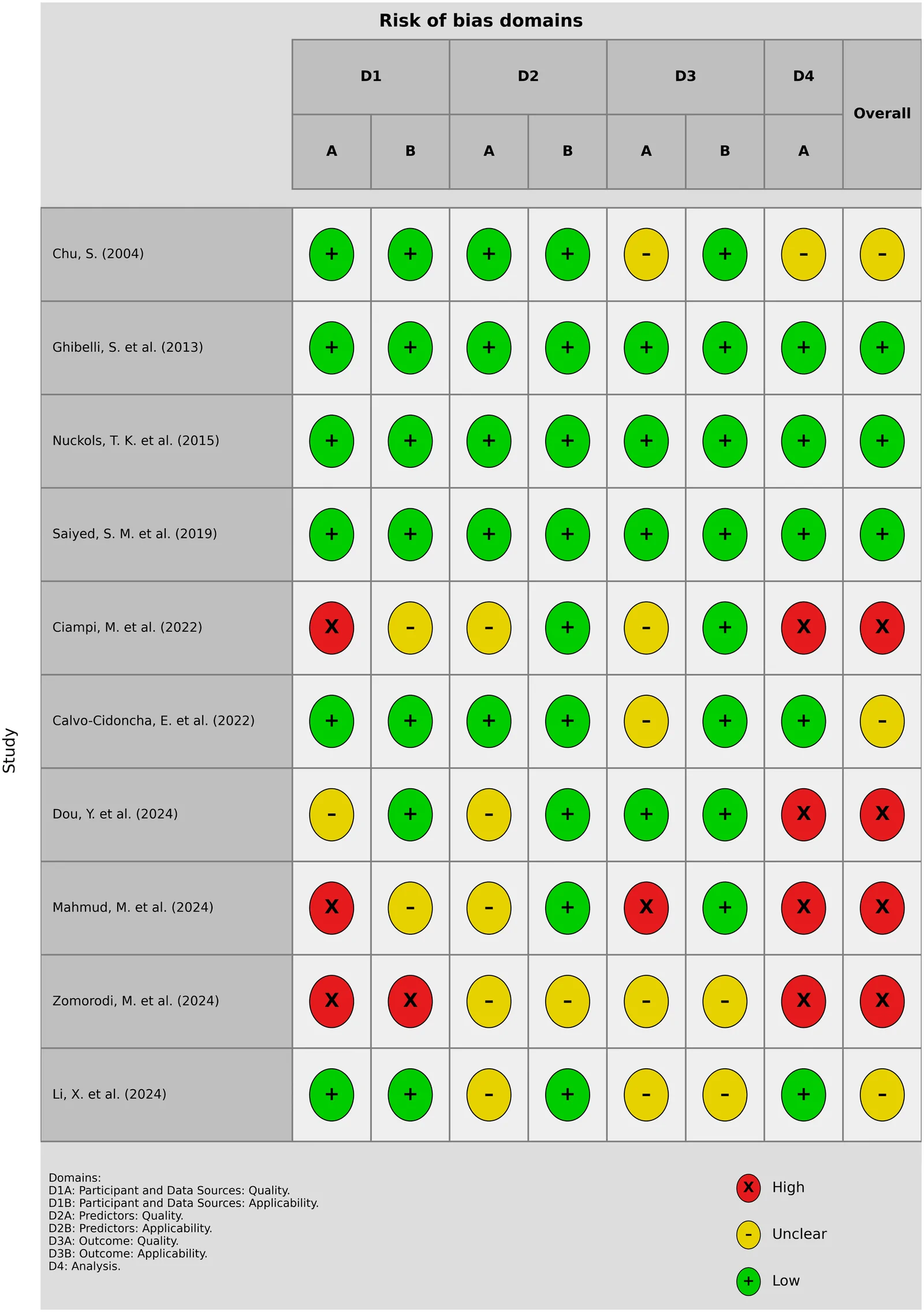
Traffic light plots depicting the risk of bias for included studies.

The PROBAST+AI asessment revealed significant divergence in risk profiles. Domain-specific and overall summary assessments are presented in Fig 2, while the detailed, study-level evaluations for all signaling questions are provided in Supporting Information, S2 Table.

The Fig 2 summarizes the risk of bias across different domains for ten studies. Considering the overall risk column, applying the worst-domain-anywhere principle, three studies (Ghibelli et al., 2013 [33]; Nuckols et al., 2015 [30]; Saiyed et al., 2019 [28]) were rated as low risk, indicating generally robust methodology and reliable results across all assessed domains. Three studies (Chu, 2004 [29]; Calvo-Cidoncha et al., 2022 [32]; Li et al., 2024 [35]) were rated as unclear, reflecting inconsistent domain-level ratings or reporting that prevents a confident judgment. Four studies (Ciampi et al., 2022 [26]; Dou et al., 2024 [27]; Mahmud et al., 2024 [31]; Zomorodi et al., 2024 [34]) were assessed as high risk, each having at least one domain, most commonly Analysis, rated high, which is sufficient under the worst-domain-anywhere principle to classify the study as high risk overall regardless of performance in other domains. Overall, the risk-of-bias profile across the included studies is mixed rather than predominantly favorable, with a notable proportion of studies, particularly those employing stochastic or generative architectures, carrying high risk of bias driven primarily by limited external validation and analysis-domain concerns.

It should be noted that a high-risk rating under PROBAST+AI reflects the current stage of a model’s clinical translational readiness rather than an inherent or permanent flaw in the underlying model itself. For most of the models rated high or unclear risk in this review, that rating is expected to improve once the clinical translational process is completed, specifically, once Domain 1 (Participants and Data Source) is addressed through representative, adequately documented patient populations, and Domain 4 (Outcome, applicability) is addressed through proper prospective clinical validation of model outputs against real-world patient outcomes. In this sense, the risk-of-bias rating reported here represents the readiness of a model for safe clinical application at the time of publication, not a negative judgment on the model’s underlying methodological or predictive validity.

### Results of syntheses

#### Early deterministic models: Detection and alerts.

Early and clinically-focused interventions framed drug-drug interaction (DDI) mitigation as a workflow optimization problem. Chu (2004) [29] demonstrated this in a pilot effort at Thomas Jefferson University Hospital. By examining 109 pharmacist interventions, Chu showed that reducing DDIs relied primarily on when pharmacist reviews were conducted, rather than on data availability alone.

Similarly, Saiyed et al. (2019) [28] addressed the “alert fatigue” phenomenon using data from CaroMont Health and The MetroHealth System. Mathematically, this is a signal detection problem: as sensitivity increases (more alerts), specificity often drops, leading to clinician desensitization. Saiyed’s work suggests that optimizing the threshold for alerts is as critical as the underlying database accuracy. On a macro scale, Nuckols et al. (2015) [30] utilized national data regarding the 2009 Health Information Technology for Economic and Clinical Health (HITECH) Act, showing that external forcing functions (policy constraints) can drive the adoption of electronic health records (EHRs), effectively standardizing the data inputs required for any DDI logic.

#### Advanced models: Ontological frameworks.

Advancing beyond simple workflow alerts, 2022 saw two influential systems built around structured, ontology-based knowledge representations for DDI mitigation, though the two differ sharply in their underlying decision-making mechanism: Calvo-Cidoncha et al. (2022) [32] implement a purely deterministic ontology, whereas Ciampi et al. (2022) [26] embed an ontological knowledge base within a broader stochastic, learning-based architecture. The OntoPharma system developed by Calvo-Cidoncha et al. attempts to map the heterogeneity of clinical notes into a rigorous ontology. This effectively reduces the dimensionality of the problem by grouping drugs into hierarchical classes.

Ciampi et al. (2022) [26] propose a home-assistance system that pairs this kind of ontological grounding with a multi-agent learning architecture, rather than relying on it as a standalone rule set. The system consists of three agents: a “Tutor” that self-learns personalised reminder timing via reinforcement learning, a “Checker” that identifies the medication being handled via deep neural networks, optical character recognition, and barcode reading, and an “Advisor” that generates DDI alerts by using natural language processing to extract active ingredients — cross-referenced against Unified Medical Language System (UMLS) [36] and RxNorm [37] resources. The pipeline as a whole adapts to the patient over time, DDI detection here functions as one stage of a learned, self-adapting ontological system.

#### Stochastic and generative systems: High-dimensional prediction.

The most recent literature (2024) abandons binary and deterministic rules entirely in favor of probabilistic modeling. Li et al. (2024) [35] employ the MIMIC-III dataset [38], which contains a dense tensor of vital signs, laboratory values, and medication records. By training generative AI systems on this de-identified dataset, Li et al. treat DDI not as a static lookup task but as a pattern-completion problem within a latent vector space.

Dou et al. (2024) [27] extend this approach by integrating large language models (GPT-4) [39] into clinical pharmacy workflows. The “Shennong-Agent” paradigm proposed by Dou et al. suggests that the model can simulate reasoning across complex interaction networks that may not be fully captured by static drug databases. Similarly, Zomorodi et al. (2024) [34] frame DDI mitigation as a recommendation-system problem, conceptually similar to algorithms used in platforms such as Netflix or Amazon. Using data extracted from Druglib.com and trained on 2,304 patients, the RECOMED system developed by Zomorodi et al. predicts the safety “rating” of candidate drug combinations.

Mahmud et al. (2024) [31] propose a hybrid architecture integrating Medical Health Records (MHR) with symptom history. This approach introduces an additional set of predictive variables, patient phenomenology, into the optimization framework, moving beyond purely chemical interaction modeling toward patient-specific interaction risk prediction.

**Fig 3:**
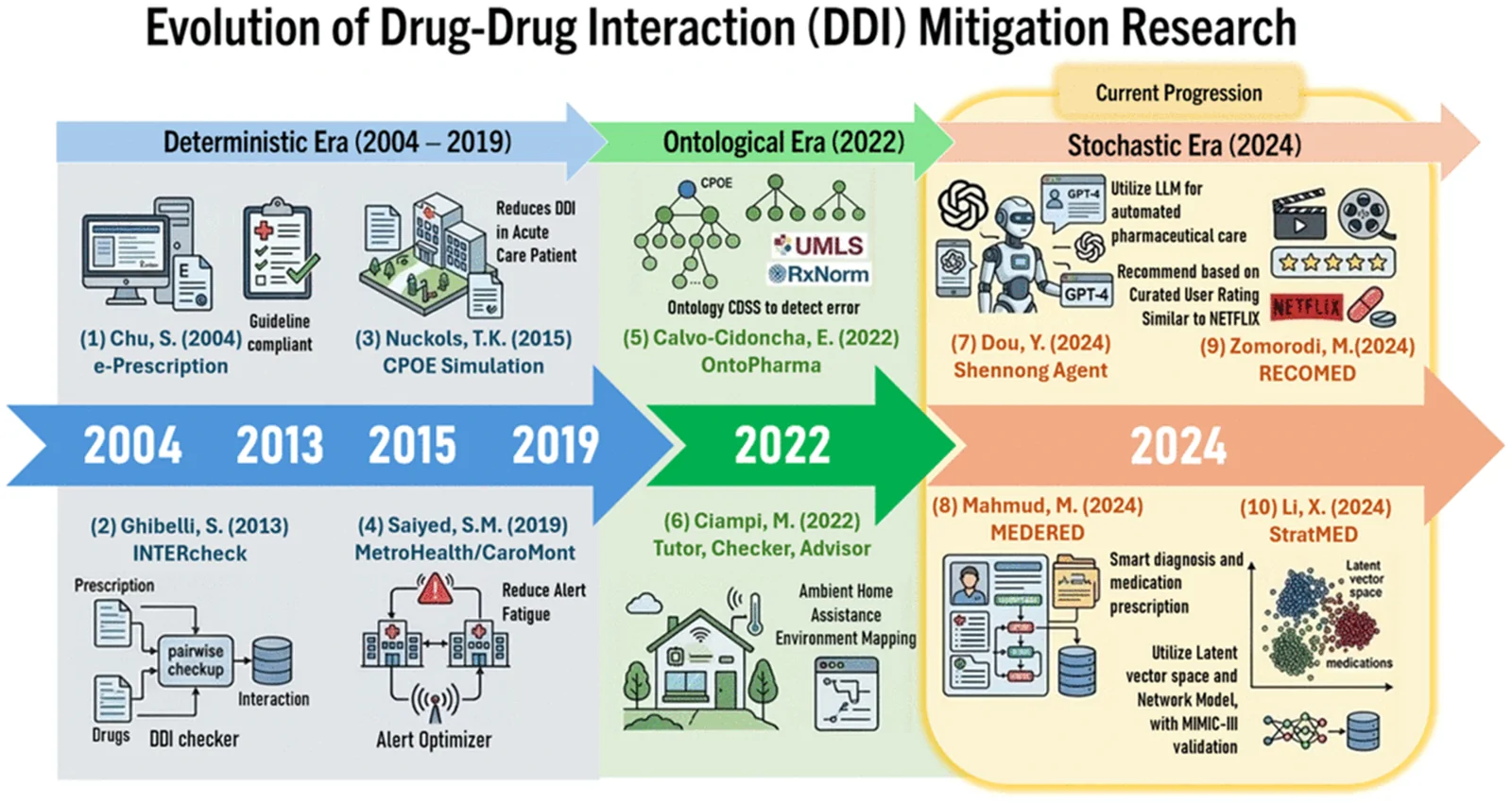
Illustration of the evolution of drug-drug interaction mitigation research.

Fig 3 shows the evolution of DDI mitigation research, progessing from deterministic, rule-based systems focused on alert generation and workflow optimization, toward ontology-driven frameworks that structure clinical knowledge into standardized semantic representations, and ultimately to stochastic, data-driven models that leverage high-dimensional data and machine learning. This progression reflects a shift from binary interaction detection to probabilistic prediction and personalized risk assessment.

## Discussion

### From deterministic rules to stochastic probabilities

The synthesis of the included literature suggests that DDI mitigation has evolved from a simple pairwise interaction lookup task into a high-dimensional prescription optimization problem. Crucially, the way these tools measure a DDI risk reduction and their comparative performance depends entirely on their underlying architecture. In contemporary clinical interpretation, prescribing risk emerges from the interaction of several factors rather than a single deterministic rule set. These include the pharmacological characteristics of the drug regimen itself, patient-specific attributes, real-time clinical phenomenology, and the institutional policy environments constraining prescribing decisions. These interacting components collectively shape the risk landscape of medication management, while stochastic variability inherent in clinical practice continues to introduce uncertainty. Early interventions, including the pharmacist-mediated oversight described by Chu (2004) [29], attempted to mitigate this uncertainty through manual verification, whereas contemporary computational approaches increasingly treat the problem as an integrated multidimensional optimization task.

### The deterministic era: Ontologies and signal optimization

The “Deterministic Era” of DDI mitigation, represented by the early clinical systems described by Chu (2004) [29] and Saiyed et al. (2019) [28], focused primarily on the human-in-the-loop. Chu (2004) highlighted that the mere presence of a CPOE system is insufficient; the timing and frequency of pharmacist interventions are the true drivers of safety. This leads to the “Signal-to-Noise” problem identified by Saiyed et al. (2019). Using data from CaroMont Health and The MetroHealth System, Saiyed et al. demonstrated that as systems become more sensitive to potential interactions, they risk crossing a threshold into “alert fatigue.” As Graham et al. (2010) [40] suggest, this cognitive desensitization effectively renders the mathematical precision of the system null if the clinician ignores the signal.

To resolve this limitation without relying on opaque probabilistic parameters, Calvo-Cidoncha et al. (2022) [32] proposed structural guardrails through an ontological framework. While this approach maximizes specificity, it lacks the flexibility required to capture patient-specific interaction dynamics observed in complex clinical contexts, such as the geriatric ward environments examined by Ghibelli et al. (2013) [33].

### The stochastic era: Probability in modeling and generative intelligence

The most significant shift identified in this review is the transition toward latent feature spaces and probabilistic modeling. An early instance of this shift is Ciampi et al. (2022) [26], whose home-assistance system embeds DDI detection within a multi-agent architecture built on reinforcement learning, deep neural networks, and natural language processing rather than a fixed rule set. Although the system’s Advisor component cross-references Unified Medical Language System (UMLS) [36] and RxNorm [37] resources in a manner reminiscent of ontological lookup, this matching step is downstream of a deep-learning-based medication identification process and operates within a self-adapting pipeline that updates its behavior based on ongoing patient interaction. This positions Ciampi et al.’s system within the stochastic paradigm, despite its partial reliance on structured knowledge bases, distinguishing it from the rigid ontological guardrails proposed by Calvo-Cidoncha et al.

Zomorodi et al. (2024) [34] and Li et al. (2024) [35] extend this shift by conceptualizing drug-drug interaction (DDI) mitigation as a recommendation problem. The RECOMED system developed by Zomorodi et al. utilizes collaborative filtering, a technique originally explored in biomedical interaction networks by Cami et al. (2013) [41], to predict previously unobserved interaction links. By training on clinical data from 2,304 patients, Zomorodi et al. effectively model drug safety as a latent rating that must be optimized across candidate drug combinations.

Li et al. extend this paradigm by incorporating high-dimensional clinical data from the MIMIC-III database [38]. In this framework, drug interactions are no longer treated as static properties of drug pairs but as dynamic states inferred from a dense tensor of patient variables, including vital signs, laboratory results, and medication histories. This transition from rule-based interaction detection to state-based prediction enables the identification of interactions that emerge only under specific physiological conditions. However, as highlighted by the contrast between the rigid ontological framework proposed by Calvo-Cidoncha et al. and the probabilistic and learning-based models developed by Ciampi et al., Li et al., and Dou et al. (2024) [26,27,35], reliability and interpretability remain important concerns. Deterministic rules, such as those implemented by Calvo-Cidoncha et al., offer transparency and safety guarantees but lack flexibility, whereas probabilistic and learning-based models, including those proposed by Ciampi et al., Li et al., and Dou et al., provide adaptive predictive capacity at the cost of potential opacity associated with black-box modeling approaches.

### Autonomous agents and the PROBAST+AI critique

The “autonomous” systems described by Dou (2024) [27] suggest a future where the error term ϵ is minimized not by human review, but by continuous reinforcement learning. Dou’s use of GPT-4 as a reasoning agent for clinical pharmacists marks the arrival of generative AI in the pharmacy domain. The PROBAST+AI analysis highlights critical vulnerabilities in the validation of these generative models. Specifically, the ’High’ risk of bias in the analysis domain is driven not only by limited external validation but also by a lack of calibration reporting [27,35]. In clinical decision support. a model’s calibration – the agreement between its predicted interaction probabilities and observed clinical event rates – is arguably more critical than discrimination metrics like the F1-score. Furthermore, models trained on highly specific datasets, such as the North American critical care standards in MIMIC-III, face significant reproducibiity challenge [38]. Without rigorous external validation across geographically and demographically diverse cohorts, the reproducibility of these high-dimensional models remains unproven, limiting their safe generalization to varied clinical settings.

Recent perspectives reinforce this concern and point toward a more collaborative alternative to full autonomy. Spanakis et al. (2026) [42] argue for a conceptual shift away from static, rule-based DDI tools toward human-augmented AI systems in which clinician feedback is embedded as an integral component of the model’s ongoing operation, rather than treated as an external check applied after the fact. This perspective suggest that for DDI-focused stochastic and generative models still lacking external validation, a collaborative human-in-the-loop architecture is a more immediately defensible deployment path than the fully autonomous agent model.

Beyond statistical validation, clinical validation demands robust explainability. As Akter and Mustafa (2024) [43] argue that the “black-box” nature of high-performance machine learning models presents a significant barrier to their clinical applicability and accountability. If a large language model (LLM)-based agent [27] advises against a prescription, the clinician requires more than a probability score; they require a transparent, explainable rationale that aligns with established medical knowledge. Akter and Mustafa demonstrate that while eXplainable AI (XAI) tools can identify key clinical indicators, a critical gap often remains between AI-derived feature importance and the “ground truth” priorities of human experts.

Finally, even a well-calibrated and explainable model faces severe implementation challenges if it disrupts the clinical workflow. As noted by Olawade et al. (2026) [44], embedding clinician oversight within the decision loop can preserve safety, but workflow integration, clinical training, and change management remain key implementation barriers. Without thoughtful implementation, highly sensitive predictive agents risk becoming sophisticated generators of ’noisy’ alerts, exacerbating the very alert fatigue they were designed to prevent.

### The case for hybrid architectures: Integrating deterministic boundaries with stochastic reasoning

The convergence of Mahmud’s symptom integration [31] and Nuckols’ policy analysis [30] suggests that the future of DDI mitigation lies in “Hybrid Architectures.” The HITECH Act [30] provided the data infrastructure, while [31] provided the clinical depth. The ultimate optimization goal is a system that uses the deterministic safety of an ontology [26] to set the boundaries, while using the generative power of a large language model [27] or a network model [34] to navigate the complexities within those boundaries.

### Heterogeneity of performance metrics

The most consistent finding in this review is that the performance metric is highly heterogeneous. The performance metrics is not merely a methodological inconvenience, it is itself the central story of DDI mitigation’s evolution. As interventions have progressed from deterministic alert logs [28,29] to ontological frameworks [26] to stochastic and generative models [27,31,34,35], each successive generation of models has required its own, increasingly specialized evaluation methodology: alert acceptance and prescribing error counts for institutional workflow systems, ontology coverage and class-mapping accuracy for semantic frameworks, and AUC, F1-score, Jaccard similarity, or RMSE for data-driven predictive models. This is not incidental. Model complexity and metric specialization have advanced, such that no single evaluation framework has kept pace across the full span of the field’s technical evolution.

This creates a structural friction that works directly against clinical translation. A model that is theoretically more advanced, and in principle capable of superior predictive performance, cannot be benchmarked against the deterministic or ontological systems it is intended to replace or augment, because there is no shared metric on which such a comparison could be made. Consequently, clinicians and health systems evaluating whether to adopt a newer, more complex model have no straightforward evidentiary basis for concluding that it performs better, in any clinically meaningful sense, than the simpler system already in place.

Because no shared outcome framework spans the deterministic, ontological, and stochastic generations of DDI-mitigation models, any cross-study comparison in this synthesis is necessarily qualitative and interpretive rather than statistical. Addressing this gap would require the field to develop a shared, translationally-oriented outcome framework, one capable of expressing both computational accuracy and clinical impact in common terms, so that genuine performance gains, where they exist, can be distinguished from advances that are novel only in their metric of measurement.

### The translational gap: Prediction performance versus patient outcome

A critical limitation identified in this review is the disconnect between computational prediction performance and measurable clinical endpoints. Early deterministic and workflow-based interventions often attempted to quantify their direct impact on patient safety. For example, Nuckols et al. (2015) [30] framed the utility of their system in terms of reduced preventable adverse drug events (PADEs) and Quality-Adjusted Life Years (QALYs) saved. Similarly, Ghibelli et al. (2013) [33] evaluated their system based on a significant reduction in potentially inappropriate medications (PIMs) and severe DDIs in clinical practice.

In contrast, the emergence of stochastic and generative models has shifted the evaluative focus almost entirely toward mathematical precision. Recent interventions, such as the network models proposed by Li et al. (2024) [35] and the predictive systems by Zomorodi et al. (2024) [34], report highly impressive internal metrics, including F1-scores, Jaccard similarity, and accuracy percentages. However, these studies do not extend their evaluations to real-world clinical environments to measure corresponding reductions in adverse drug events (ADEs) or patient morbidity.

Consequently, the current body of literature does not provide sufficient evidence to conclude that improvements in DDI prediction performance directly lead to better patient outcomes. This disconnect represents the core of the “last mile” problem in medical informatics. Until stochastic and AI-driven models are subjected to prospective clinical trials that measure tangible patient safety outcomes–rather than just algorithmic accuracy–their ultimate clinical value remains theoretical.

### Limitations of the evidence and review process

The primary limitation of this review is precisely what precludes a direct, quantitative comparison of DDI-related case reduction across differing prediction-performance levels. For example, the performance of a 2004 deterministic-era intervention cannot be quantitatively compared to a 2024 generative LLM using a single meta-analytic effect size. The former reports clinical process indicators (e.g., alert acceptance), while the latter relies on computational accuracy metrics (e.g., AUC, F1-score). This profound methodological heterogeneity is precisely what prevents a direct comparison of DDI-related case reduction. Furthermore, the reliance on datasets like MIMIC-III [35] introduces a geographical bias toward North American critical care standards, which may not be directly comparable to the geriatric wards of Northern Italy [33] or home care contexts in Europe.

### Implications for practice and future research

Future research must prioritize the development of “Explainable Models” for stochastic computations, including AI systems, with a solid, translatable benchmark. As argued above, the field’s central obstacle is not a lack of algorithmic sophistication but the absence of a shared evaluation standard that spans deterministic, ontological, and stochastic architectures alike; without such a benchmark, a newer model’s theoretical advantage cannot be demonstrated in terms that are meaningful relative to the ontological or rule-based systems already in clinical use. Critically, this benchmark needs no, and likely should not, be purely numerical. Rather than relying solely on statistical accuracy parameters such as AUC or F1-score, a translatable benchmark could instead be built around explainability, safety, and reasoning, evaluating whether a model can articulate why a given prescribing decision was reached, whether that reasoning aligns with established clinical and ontological safety constraints, and whether its outputs remain within the deterministic safety boundaries already trusted by clinicians. Such a benchmark would allow deterministic, ontological, and stochastic systems to be compared on a common, clinically interpretable footing, rather than on disjoint numerical scores that carry no shared meaning across model generations. Patient-safety-level clinical validation, the gold standard by which such a benchmark would ultimately need to be confirmed, is difficult to apply at this stage, as it requires prospective human testing, and most clinical institutions are understandably hesitant to expose patients to a stochastic or generative model before that model has first demonstrated, on some shared and interpretable footing, that it performs at least as safely as the deterministic and ontological guardrails it would sit alongside or replace. This allows the system to predict and reason while remaining mathematically constrained from suggesting combinations that violate core safety ontologies established by clinical guidelines. Until AI models can withstand the scrutiny of clinical validation requirements, their role should remain supportive rather than autonomous.

## Conclusion

The systematic review of these ten pivotal studies illustrates a mathematical and clinical trajectory from reactive, deterministic intervention to proactive, stochastic modeling. Given the limited pool of included studies, it is difficult to determine whether the technical performance gains reported by AI-driven models translate into a proportional, measurable improvement in clinically reliable decision support. The domain has matured from the localized, pilot monitoring of pharmacist-led interventions [29] to the deployment of high-dimensional autonomous agents capable of parsing vast medical ontologies and latent physiological patterns [27, 35]. This evolution reflects a broader shift in health informatics: moving beyond simple Boolean checks toward a “learning health system” architecture where drug-drug interaction (DDI) mitigation is treated as a continuous optimization problem.

However, the “perfect” system remains computationally and clinically elusive. While the integration of high-fidelity datasets such as MIMIC-III [38] and standardized lexicons such as RxNorm has significantly empowered algorithmic precision, clinical utility remains bottlenecked by the “last mile” problem, the gap between mathematical probability and clinician actionability. Institutional workflow interventions [28,29] and AI-driven predictive models [27,34] report fundamental different construct of performance, clinical process indicators and technical accuracy metrics, respectively, and this incompatible measurability means their outcome cannot be directly compared.

Ultimately, the transition from rigid rules to fluid generative reasoning appears to introduce a potential new risk profile characterized by algorithmic opacity and the possibility of hallucination. To address these limitations, future research should consider focusing on the hybridization of these methodologies. The rigorous external validation of stochastic models to address the “High” analysis bias identified in this review, and the seamless integration of these predictions into the clinical workflow to mitigate the issue of alert fatigue [28]. This synthesis of mathematical optimization and human-centric design that the reduction of inappropriate prescribing translates into tangible, reproducible patient safety.

## Supporting information

S1 Table PRISMA Checklist. (PDF)

S1 File List of included studies from title and abstract screening. (XLSX)

S2 File List of excluded studies with reasons. (XLSX)

S2 Table PROBAST+AI assessment. (PDF)
