# Supplementary material for: Deterministic and stochastic interventions in reducing drug–drug interactions in inappropriate prescribing: A systematic review
Source: PLoS One. 2026 Sep 17;21(9):e0356883. doi: 10.1371/journal.pone.0356883 (PMC13585254; doi:10.1371/journal.pone.0356883)
Supplement: Supplementary file 2 [file pone.0356883.s004.pdf]

	Models of interest	AMICO	Shennong	Epic CDS	ePrescribe	CPOE	MEDERED	OntoPharma	INTERcheck	RECOMED	StratMed
	Outcome of interest	Errors	Safety	Alerts	Usability	Costs	Accuracy	Alerts	PIMs	Hit Rate	DDI Rate
	PROBAST+AI: MODEL DEVELOPMENT										
DOMAIN 1:	Participants and data sources										
A.	Quality										
1.1	Were appropriate data sources used?	NI	NI	PY	NA	PY	NI	PY	Y	PN	PY
1.2	Was an appropriate study design used?	PN	PY	PY	NI	Y	PN	PY	PY	PY	PY
1.3	Did the in- and exclusions of study participants result in a representative dataset?	N	NI	PY	NA	PY	N	PY	Y	NI	PY
	Concern regarding quality of selection of participants and data sources	high	unclear	low	low	low	high	low	low	high	low
	Rationale of quality rating:	Participant data and clinical sampling procedures are not detailed; focus is on system architecture and experimental algorithm performance rather than a representative clinical dataset	While the data for geriatric hospital is stated, no information of the trained LLM dataset	Comprehensive alert metadata was used across all categories and care settings	No patient participant sampling; primary focus is system specification and pilot discussion, a pilot study	The model does not use a clinical cohort but reputable literature-derived parameters; risk stems from heterogeneity and assumptions in published inputs.	The model is built on conceptual integration of an MHR and external dataset references (e.g., PubMed extraction) but lacks detailed clinical dataset sourcing.	Clear inclusion of relevant clinical data in a real hospital setting supports reliability, though limited to a single unit.	Clinical prescribing data were included for all eligible geriatric patients within defined periods.	Datasets are synthetic or aggregated from drug databases and rating sources without detailed clinical sampling frames; thus representativeness for clinical prescribing populations is uncertain.	Data are real clinical records but constrained to one EHR dataset
B.	Applicability										
	Concern that the (data of the) included participants do not match the review question or the assessor's intended use of the prediction model	unclear	low	low	low	low	unclear	low	low	high	low
	Rationale of applicability rating:	The model states the use of continuous monitoring that used as data	Data approved by ethics board	Alert metadata from real clinical settings directly matches the intent to assess strategies to optimize real EHR CDS	Although not a clinical study, the system is designed for real prescribing workflows at the point of care.	The population (hospitalized patients) and outcomes (costs, health outcomes) align with policy evaluation of CPOE implementation.	The intended clinical application matches prescription error reduction in healthcare, but absence of actual clinical participants limits assessment of representativeness.	The CDSS is applied to real prescribing events in typical internal medicine patients, aligned with intended clinical use.	Target population (hospitalized elderly patients with polypharmacy) directly matches the review question and intended use of the CDSS.	The constructed dataset may not reflect clinical populations or real prescribing scenarios; applicability to real patient care is limited without clinical validation.	Study data directly relate to medication prescribing behaviors in clinical EHR, aligning well with intended medication recommendation.
DOMAIN 2:	Predictors										
A.	Quality										
2.1	Were predictors defined and assessed in a similar way for all participants?	NI	NI	PY	NI	PY	NI	PY	Y	PY	Y
2.2	Was any pre-processing of predictors similar for all participants?	NI	NI	PY	NI	PY	NI	PY	Y	Y	Y
2.3	Were predictor assessments made without knowledge of outcome data?	NI	NI	NA	NA	NA	NI	NI	NA	N	N
2.4	Were the predictors included in the model available at the time the model was intended to be used?	Y	PY	Y	NA	NA	PY	Y	Y	PY	Y
	Concern regarding the quality of the predictors or their assessment	unclear	unclear	low	low	low	unclear	low	low	unclear	unclear
	Rationale of quality rating:	Predictors are practical for home settings, but definitions and consistency of assessment across users are not thoroughly described	Predictors are implicitly clinical and medication-related information accessible at care time, but detailed definitions and handling are not reported.	Alert metrics are directly measurable from EHR reporting	Predictors are irrelevant since no predictive model is developed.	Inputs reflect literature but are subject to variability and uncertainty inherent in secondary data.	Lack of reporting on predictor definitions and assessment procedures.	Essential clinical predictors are integrated into CDSS alert logic.	Standardized medical data informed the support system across all subjects.	Predictors are computationally derived; real-world data integration and clinical relevance require further validation.	Predictors are standard but use deep embeddings that may obscure interpretability.
	Applicability										
	Concern that the definition, pre-processing, assessment, or timing of assessment of the predictors in the model do not match the review question or the assessor's intended use	low	low	low	low	low	low	low	low	unclear	low
	Rationale of applicability rating:	Predictors align with real-world medication use and error detection tasks.	Medication and clinical data used by LLMs align with real-world pharmaceutical care workflows.	EHR alert metrics match intended evaluation of alert optimization	Not applicable	Parameters used (costs, event probabilities) are relevant to societal cost-utility context.	Predictors align theoretically with clinical prescription contexts.	Ontology captures clinically relevant characteristics needed for safe prescribing.	Clinical prescribing data are precisely what the CDSS is designed to monitor.	Predictors are relevant for algorithmic recommendation but not validated within clinical prescribing workflows.	Real clinical features used closely resemble what would be available for a clinical decision support system.
DOMAIN 3:	Outcome										
A.	Quality										
3.1	Were outcomes defined and assessed appropriately?	PY	PY	Y	PN	Y	PY	PY	Y	PY	Y
3.2	Were outcomes defined and assessed in a similar way for all participants?	NI	NI	Y	NI	Y	NI	Y	Y	Y	Y
3.3	Were outcome assessments made without use or knowledge of predictor data?	N	N	NA	NA	NA	N	N	N	N	N
3.4	Was the time interval between predictor assessment and outcome assessment appropriate?	Y	PY	Y	NA	Y	NI	Y	Y	NA	NA
	Concern regarding quality of the outcome or its determination	unclear	low	low	high	low	high	unclear	low	unclear	unclear
	Rationale of quality rating:	The algorithm performance is properly presented (hyperparameters and learning curve), but not the measured patient safety outcome	Outcomes are conceptually appropriate but not operationalized or independently assessed; LLM reasoning inherently uses predictor data.	Alert count measures are objective and consistent.	No clinical measurement of outcomes; benefits are speculative or descriptive.	Outcomes are standard economic endpoints, not subject to subjective or inconsistent measurement.	Outcomes are preliminary prototype results on accuracy without clinical validation.	Although outcomes are well described, clinical effectiveness (actual error reduction) is not independently measured—acceptance rate is a surrogate.	Outcomes are directly meaningful and consistently assessed.	Performance metrics are computationally valid but do not substitute clinical outcomes.	Metrics reflect algorithmic performance but do not measure clinical outcomes (e.g., reduced adverse events).
B.	Applicability										
	Concern that the outcome, its definition, assessment, or timing of assessment do not match the review question or the assessor's intended use	low	low	low	low	low	low	low	low	unclear	unclear
	Rationale of applicability rating:	Outcome of preventing medication errors at home is directly relevant to the intended use	Medication safety and pharmaceutical care quality are appropriate and clinically relevant outcomes	Alert metrics directly quantify intended CDS performance improvements	Conceptual outcomes (improve prescribing and safety) match intent	Outcomes directly address policy questions about cost-effectiveness and societal benefit of CPOE	Error reduction accuracy is relevant to prescription safety purposes	Alerts and their acceptance are relevant measures of safe prescribing support	Outcome measures directly relate to prescribing appropriateness reduction goals	Model performance metrics may not directly reflect clinical prescribing success or safety; clinical endpoints are not measured	Algorithm performance does not directly equate to improved patient outcomes without clinical validation
